# Editorial: Immunomodulation by vitamins: mechanistic insights and clinical translation

**DOI:** 10.3389/fimmu.2026.1981352

**Published:** 2026-09-09

**Authors:** Dieter Kabelitz

**Affiliations:** Institute of Medical Immunology, University of Kiel and University Hospital Schleswig-Holstein, Kiel, Germany

**Keywords:** gamma/delta T cells, inflammation, retinoic acid, septic shock, trace elements, vitamin C, vitamin D

Micronutrients including vitamins and trace elements play essential roles in the regulation of immune responses in health and disease. They are required to maintain immune homeostasis and are therapeutically used in inflammatory disorders and chronic diseases including cancer (1). At the molecular level, vitamins exert pleiotropic activities. For example, vitamin C combines antioxidant activity with essential functions as co-factor for several enzymes (e.g. monooxidases and iron-dependent or 2-oxoglutarate-dependent dioxygenases) and epigenetic regulator (2). Vitamin C is essential for proper assembly of collagen fibers, and severe Vitamin C deficiency is linked to the scurvy syndrome (3). Furthermore, Vitamin C modulates T-cell activation and differentiation and functionally supports Treg activity through demethylation of critical regions in the *foxp3* gene, the master transcription factor of Treg (4). Similarly, Vitamin D also exerts multiple effects on the immune system, in addition to its metabolic role in balancing the calcium and phosphate levels (5).

In this Research Topic, selected aspects of vitamins are discussed with respect to molecular immune regulation and clinical translation. Specifically, *in vitro* effects of the vitamin A derivative retinoic acid and of vitamin C are studied on inflammatory cytokine induction in human macrophages and on human γδ T-cell activation, respectively. Translational studies analyze the serum levels of multiple vitamins in children with *Mycoplasma pneumoniae* pneumonia and the potentially beneficial effects of vitamins in patients with septic shock. Two additional reviews address the role of vitamin D in the gastrointestinal tract and in autoimmune thyroid disease.

Ahmad et al. studied the modulation of pro- and anti-inflammatory cytokine induction in human macrophage subsets by all-trans retinoic acid (ATRA), the vitamin A derivative which functions as a ligand for the retinoic acid receptor. They differentiated human monocytes into pro-inflammatory M1 or anti-inflammatory M2 macrophages and used ligands of cytosolic pattern-recognition receptors NOD1 and NOD2 for cytokine induction. The results indicated that ATRA differentially modulates NOD1 and NOD2-induced secretion of several cytokines, and the effect of ATRA also depends on the differentiation status of the macrophage subset. The authors conclude that the retinoic acid concentration in the local tissue environment may critically shape the functional plasticity of macrophage subsets.

In contrast to conventional αβ T cells, γδ T cells are usually not MHC-restricted. The major subset of human γδ T cells recognize pyrophosphate metabolites of microbial or tumor cell origin, and tumor-reactive γδ T cells have raised substantial interest as effector cells in cancer immunotherapy (6, 7). It has been previously reported that vitamin C enhances the effector functions of human γδ T cells *in vitro* (8), and vitamin C-expanded γδ T cells have already been adoptively transferred into cancer patients (9). However, the molecular pathways how vitamin C co-stimulates γδ T-cell activation have not yet been investigated. This topic was addressed by Zarobkiewicz et al. in their contribution to this Research Topic. They observed that vitamin C enhanced tumor cell killing not only by unmodified γδ T cells but also by γδ T cells engineered to express synthetic antigen receptors. Moreover, vitamin C increased calcium influx upon T-cell receptor activation and modulated downstream signaling pathways at the level of ERK1/2 activation and nuclear translocation of NFAT and NF-κB. These results advance our understanding how T-cell receptor signaling mechanisms are affected by vitamin C.

In a retrospective observational study, Li et al. observed lower serum concentrations of several vitamins (A, D, B1, B7, C) in a cohort of 135 children with *Mycoplasma pneumoniae* pneumonia compared to the non-infected control group. Multivariate regression analysis confirmed that the vitamin serum levels were independently associated with *Mycoplasma pneumoniae* pneumonia in this single center study. As the authors rightly point out, future large-scale prospective multi-center studies are required to verify a potential link between serum vitamin deficiency and *Mycoplasma pneumoniae* pneumonia.

Vitamin supplementation has been used as adjunctive therapy in critically ill patients with sepsis or septic shock, but inconsistent results have been reported. Tian et al. performed a Bayesian network meta-analysis based on 36 articles reporting efficacy and safety of randomized controlled trials with various vitamins, also in combination with other modalities, in critically ill patients. Overall, it appeared that the use of vitamin D had a beneficial effect on length of stay in the intensive care unit and overall hospitalization, but none of the interventions had a significant effect on mechanical ventilation time or 28-day mortality.

Vitamin D is also the topic of the review by Sonsalla et al. The authors summarize the role of vitamin D in the gastrointestinal tract and relevant clinical studies. The nuclear vitamin D receptor (VDR) is strongly expressed in the gastrointestinal tract and regulates intestinal epithelial barrier function but also impacts on the local immune system and indirectly on the gut microbiome. Vitamin D supplementation might be beneficial in IBD patients, and the authors raise the interesting question whether luminal delivery routes might be more effective than systemic application in these patients.

In a final contribution, Li et al. review the role of vitamin D and trace elements in autoimmune thyroid diseases like Grave’s disease and Hashimoto’s thyreoditis. While clinical studies suggest a higher prevalence of vitamin D deficiency in patients, it is difficult to exclude a role of confounding factors in such studies. The review also summarizes (mainly observational) studies on the role of trace elements like iodine, selenium, zinc, iron, copper and magnesium in autoimmune thyroid diseases.

Vitamins are essential for health and vitamin deficiency may cause or contribute to serious diseases. This Research Topic contributes to our understanding of molecular mechanisms and clinical significance of selected vitamins.
